# Entwicklung der Zirkumzisionszahlen in Deutschland seit Billigung der rituellen Beschneidung: Eine bevölkerungsbezogene Analyse von 2013 bis 2018

**DOI:** 10.1007/s00120-023-02104-6

**Published:** 2023-05-31

**Authors:** Cem Aksoy, Aristeidis Zacharis, Christer Groeben, Philipp Karschuck, Luka Flegar, Martin Baunacke, Christian Thomas, Marcel Schmidt, Johannes Huber

**Affiliations:** 1 Klinik für Urologie, Philipps-Universität Marburg, Baldingerstr., 35033 Marburg, Deutschland; 2 Klinik und Poliklinik für Urologie, Medizinische Fakultät Carl Gustav Carus, Technische Universität Dresden, Dresden, Deutschland; 3 Coloplast GmbH, Hamburg, Deutschland

**Keywords:** Zirkumzision, Phimose, Beschneidung, Beschneidungsgesetz, Religion, Circumcision, male, Phimosis, Foreskin, Law, Religion

## Abstract

**Einleitung:**

Die Zirkumzision Minderjähriger aus kulturellen und religiösen Gründen ist umstritten. Bei der Diskussion zum Beschneidungsgesetz von 2012 wurde eine Zunahme der Zirkumzision Minderjähriger ohne medizinische Indikation befürchtet. Ziel der Arbeit war es daher, die Entwicklung der Fallzahlen zu analysieren.

**Material und Methoden:**

Auf Basis der Forschungsdatenbank des Instituts für angewandte Gesundheitsforschung GmbH (InGef) mit nach Alter und Region repräsentativen 4,9 Mio. Versichertenanonymen schätzten wir die jährlichen Zirkumzisionszahlen in Deutschland von 2013 bis 2018. Dabei stratifizierten wir die Daten nach Alter (< 18 vs. ≥ 18 Jahren), Kassenärztlicher Vereinigung und Art der Leistungserbringung (ambulant vs. stationär).

**Ergebnisse:**

Im Studienzeitraum wurden insgesamt 673.819 Beschneidungen durchgeführt. Ab 2014 kam es zu einem signifikanten Rückgang der Fallzahlen in allen Altersgruppen (*p* = 0,049). Dabei haben während des gesamten Studienzeitraums die Beschneidungen bei Minderjährigen signifikant zugenommen (*p* = 0,002) und die Eingriffe bei Erwachsenen signifikant abgenommen (*p* = 0,01). Die Zahl der männlichen Minderjährigen stieg um 4 % von 6.709.137 (2013) auf 6.992.943 (2018). Die entsprechende bevölkerungsbezogene Zahl stieg von 7,5 Beschneidungen pro 1000 Minderjährige im Jahr 2013 auf 8 im Jahr 2018 (*p* = 0,037).

**Schlussfolgerung:**

Nach der Verabschiedung des Beschneidungsgesetzes 2012 kam es zu einer moderaten Zunahme der Zirkumzisionen in der Altersgruppe < 18 Jahre. Eine Limitation unserer Studie ist, dass ein unbestimmter Anteil ritueller Beschneidungen außerhalb des Gesundheitssystems erfolgte.

## Abstract (en)

**Introduction:**

The religious and cultural circumcision of male infants in Germany is controversially discussed. After the passing of the religious circumcision bill in 2012, an increase of infant circumcisions without medical indication was feared. The aim of this study was to analyze the development of the circumcision case numbers.

**Materials and methods:**

We used the research database of the German Institute for Applied Health Research with a representative anonymous sample of 4.9 million insured persons to estimate the annual circumcision numbers in Germany from 2013–2018. We stratified the data according to age (< 18 vs. ≥ 18 years). The number of male adolescents in the study period was taken from the database of the German Federal Statistical Office.

**Results:**

In the study period, 673,819 circumcisions were performed. From 2014, there was a significant decrease in the number of cases across all age groups (*p* = 0.049). Thereby, circumcisions in minors significantly increased (*p* = 0.002) and procedures in adults significantly decreased (*p* = 0.01) during the entire study period. The number of male minors increased by 4% from 6,709,137 (2013) to 6,992,943 (2018). The corresponding population-based number increased from 7.5 circumcisions per 1000 minors in 2013 to 8 in 2018 (*p* = 0.037).

**Conclusions:**

After the passage of the circumcision bill in 2012, there was a significant increase of circumcisions in the age group of < 18 years in Germany. A major limitation of our study is that presumably many ritual circumcisions might not be provided within the health care system.

## Einleitung

Während die männliche, neonatale Zirkumzision in den USA von der Amerikanischen Akademie für Pädiatrie (American Academy of Pediatrics, AAP) aufgrund möglicher gesundheitlicher Vorteile als präventive Maßnahme empfohlen wird, ist sie in Deutschland bei jungen Männern kein medizinischer Standard [1]. Die deutsche S3-Leitlinie „Diagnostik, Therapie und Nachsorge des Peniskarzinoms“ benennt zwar den Vorteil für die Prävention des Peniskarzinoms, empfiehlt die Zirkumzision jedoch nicht generell [2].

Medizinisch indiziert ist die Zirkumzision u. a. bei der Phimose, der chronischen Balanitis, dem Lichen sclerosus, Präkanzerosen und dem Plattenepithelkarzinom der Vorhaut. Unabhängig von medizinischen Vor- und Nachteilen ist die männliche genitale Beschneidung seit langer Zeit Teil vieler kultureller und religiöser Bräuche. Bereits 2300 Jahre v. Chr. wurden im alten Ägypten Zirkumzisionen durchgeführt [3]. Darüber hinaus gehört die Zirkumzision minderjähriger Männer im Judentum als auch im Islam zum festen Bestandteil der Religion [4]. Auch im Christentum findet die Zirkumzision männlicher Minderjähriger Anwendung in einigen afrikanischen Kirchen [5]. Relevante Komplikationen von Zirkumzisionen traten in einer Metaanalyse mit ca. 4 Mio. Eingriffen in 3,8 % auf [6]. Eine systematische Übersichtsarbeit zu sexuellen Auswirkungen bei männlichen Zirkumzisionen zeigte minimale bis keine nachteilige Auswirkungen und sogar teilweise in einigen Studien positive Effekte auf die sexuelle Funktionen [7]. Aufgrund der irreversiblen Veränderung im Intimbereich und möglicher Komplikationen auch die Psyche betreffend, wird die rituelle Zirkumzision in Deutschland sehr kontrovers diskutiert. Medizinethische Aspekte wie u. a. die Berücksichtigung des Selbstbestimmungsrecht des Kindes, das Kindeswohl und der Schutz der Intimsphäre sind hierbei zentral [8].

Vor dem Hintergrund der fehlenden Rechtssicherheit in Deutschland musste die Zulässigkeit der rituellen Zirkumzision 2012 gesetzgeberisch geklärt werden. Bei der Diskussion zum Beschneidungsgesetz wurde eine Zunahme der Zirkumzision von Minderjährigen ohne medizinische Indikation befürchtet. Ziel dieser Arbeit war es daher, die Entwicklung der Zirkumzisionsfallzahlen bei Minderjährigen nach Verabschiedung des Beschneidungsgesetzes am 12. Dezember 2012 zu analysieren.

## Material und Methoden

Die tatsächliche Zahl ritueller Zirkumzisionen lässt sich in Deutschland prinzipiell nicht erfassen, da diese offiziell außerhalb des Gesundheitswesens erfolgen oder als nicht medizinisch indizierte Zirkumzision in den Bereich der Individuellen Gesundheitsleistung (IGeL) fallen. Allerdings weist die betroffene Altersgruppe sehr häufig eine Phimose auf und es besteht damit eine relative medizinische Indikation zur Zirkumzision. Bei auch aus kulturellen Gründen gewünschter Zirkumzision, wird die Indikation zum Eingriff in der Regel gestellt werden. Damit dürfte ein relevanter Anteil der kulturell gewünschten Zirkumzisionen als ärztlicher Eingriff aufgrund einer vorliegenden Phimose erfolgen. Auf Basis dieser Überlegung nutzten wir die Zirkumzisionsdaten der Betriebs- und Innungskrankenkassen als Surrogatparameter zur Bestimmung der Fallzahlentwicklung ritueller Zirkumzisionen. Die Forschungsdatenbank des Instituts für angewandte Gesundheitsforschung Berlin GmbH (InGef) enthält Daten der Inanspruchnahme von ca. 7,2 Mio. anonymisierten Daten von Versicherten der Jahre 2013 bis 2018 aus mehr als 60 Betriebs- und Innungskrankenkassen aller Regionen der Bundesrepublik Deutschland. Hieraus verwendeten wir eine repräsentative Stichprobe von 4,9 Mio. Versichertenanonymen, gemäß der Alters- und Geschlechtsverteilung, um die jährlichen Zirkumzisionszahlen in Deutschland hochzurechnen. Die Zirkumzision definierten wir durch die Operationen- und Prozedurenschlüssel (OPS) 5‑640.2 und 5‑640.3. Diese Schlüssel kodieren die Zirkumzision (OPS-Kode: 5‑640.2) sowie die Frenulum- und Präputiumplastik (OPS-Kode: 5‑640.3), da diese gleichzeitig bei radikalen Zirkumzisionen durch niedergelassene Ärzt*innen sowie Ärzt*innen im Krankenhaus nach § 115b SGB V als Prozedur eingetragen werden. Dabei stratifizierten wir die Daten nach dem Patientenalter (< 18 vs. ≥ 18 Jahren). Die Anzahl männlicher Jugendlicher im Studienzeitraum entnahmen wir der Datenbank des Statistischen Bundesamtes (Destatis) für Bevölkerungsdaten. Hieraus erstellten wir sodann eine Hochrechnung aller durchgeführten Zirkumzisionen im untersuchten Zeitraum für Deutschland und stellten diese grafisch dar. Da die verwendeten Daten anonymisiert vorlagen, war kein zusätzliches Ethikvotum erforderlich. Trends über den Zeitverlauf prüften wir mittels linearer Regressionsanalyse (Signifikanzniveau *p* = 0,05). Die prozentualen Angaben beziehen sich auf die ganze untersuchte Stichprobe. Die statistische Auswertung wurde mit IBM SPSS Statistics 27 (Armonk, NY, USA) durchgeführt.

## Ergebnisse

**Figure 1:**
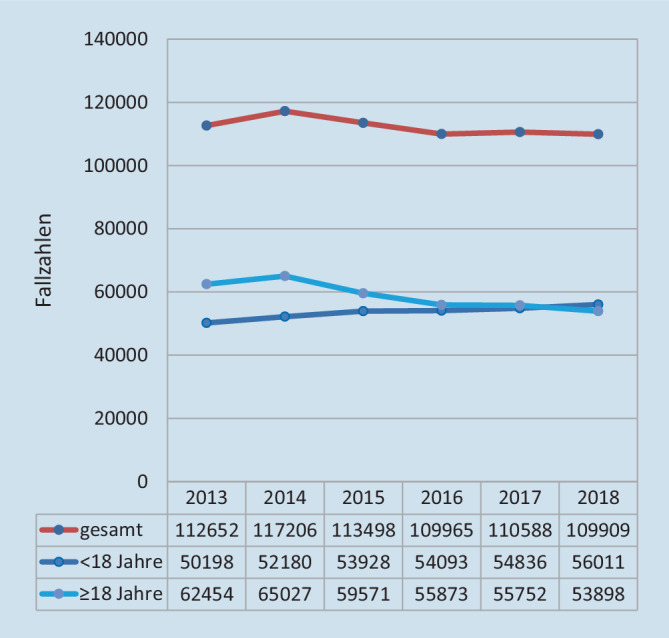

Von 2013 bis 2018 wurden in Deutschland insgesamt 673.819 Zirkumzisionen durchgeführt. Der Anteil ambulanter Eingriffe lag im Studienzeitraum konstant bei 89 %. Ab 2014 kam es über alle Altersgruppen zu einem signifikanten Rückgang der Fallzahlen (*p* = 0,049). Dabei nahmen die Zirkumzisionen bei Minderjährigen während des gesamten Studienzeitraums signifikant zu (*p* = 0,002) und die Eingriffe bei Erwachsenen signifikant ab (*p* = 0,01; Abb. 1).

**Figure 2:**
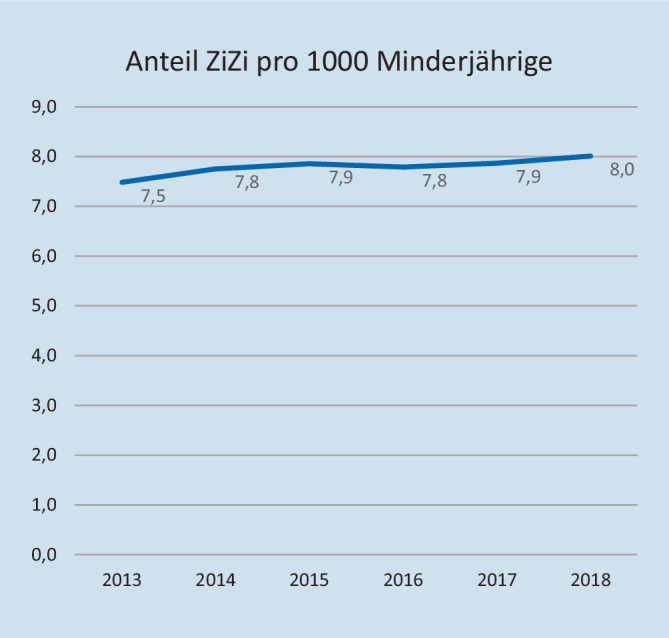

Die bundesweite Anzahl männlicher Minderjähriger nahm im Studienzeitraum zu. Sie stieg von 6.709.137 (2013) auf 6.992.943 (2018) um 4 %. Die entsprechend bevölkerungsbezogene Anzahl stieg von 7,5 Zirkumzisionen pro 1000 Minderjährigen im Jahr 2013 auf 8 im Jahr 2018 (*p* = 0,037). Auch in Bezug auf die wachsende Population männlicher Minderjähriger kam es damit zu einer signifikanten Zunahme an Zirkumzisionen (Abb. 2).

## Diskussion

Nach Billigung der rituellen männlichen Beschneidungen im Dezember 2012 kam es zu einer moderaten Zunahme der Beschneidungen männlicher Minderjähriger in Deutschland, während der Anteil bei Erwachsenen Zirkumzisionen im gleichen Zeitraum sank.

Dieser Trend der Zunahme an Zirkumzisionen männlicher Minderjähriger zeigte sich teilweise auch im internationalen Vergleich. In den USA kam es in den Jahren 2012 bis 2017 zu einer signifikanten Zunahme an männlichen neonatalen Zirkumzisionen [1]. Grund für diese Entwicklung in den USA kann die aktualisierte Stellungnahme der AAP zu neonatalen Zirkumzisionen im Jahr 2012 sein [9]. Die vorherige Stellungnahme der AAP von 1999 zu männlichen neonatalen Zirkumzisionen in den USA war deutlich neutraler formuliert [10]. Eine Analyse in den Jahren der neutraleren Stellungnahme der AAP zu neonatalen männlichen Zirkumzisionen von 1998 bis 2011 zeigte einen Rückgang der männlichen Beschneidung von Neugeborenen entsprechend der damaligen medizinischen Richtlinienempfehlung [11]. Eine Studie von Jacobson et al. fand zusätzlich heraus, dass zwischen den Jahren 2003 und 2016 die Beschneidung von Neugeborenen bei privat versicherten Neugeborenen mit 65 % signifikant höher war als bei gesetzlich versicherten Neugeborenen mit 45 % [12]. Eine mögliche Erklärung hierfür wäre, dass in den USA Menschen mit einem höheren Einkommen und einem in der Regel damit verbundenem höherem Ausbildungsstand die Zirkumzision ihres neugeborenen Kindes stärker präferieren. Ein anderer Trend der Zirkumzision männlicher Minderjähriger hingegen zeigt sich in Südkorea. Hier betrug nach einer Studie von Kim et al. die altersstandardisierte Beschneidungsrate für südkoreanische Männer im Alter von 14–29 Jahren 76 % (zwischen 2009–2011), während die Beschneidungsrate im Jahr 2002 für die gleiche Altersgruppe 86 % betrug [13]. Die Autorengruppe erklärte sich den Rückgang der Zirkumzisionszahlen in Südkorea durch die Wissenszunahme der koreanischen Bevölkerung über die Beschneidung und die damit verbundenen Vor- und Nachteilen. In Südkorea führte die postulierte stärkere Informiertheit der Bevölkerung zu einem deutlichen Rückgang der Eingriffszahlen.

Unabhängig von der Zu- und Abnahme männlicher Beschneidungen sei laut einer Studie von Hassan et al. die Zirkumzision männlicher Minderjähriger ein sicherer Eingriff ohne größere Komplikationen, wenn dieser von geschultem medizinischem Personal unter aseptischen Bedingungen im Operationssaal durchgeführt wird [14]. Die Autoren untersuchten die männlichen Beschneidungen aus religiösen Gründen in einem muslimisch geprägten Teil Indiens und beschrieben, dass Beschneidungen durch fachlich nicht qualifizierte Personen und die damit verbundenen Komplikationen in der indischen Gesellschaft immer noch weit verbreitet sind.

Für Deutschland lag bislang nur eine einzige Studie vor, welche die Entwicklung der Zirkumzisionen nach Billigung der rituellen Zirkumzisionen analysierte. Die analysierten Daten enthalten jedoch ausschließlich stationäre Eingriffe, die einen Rückgang der stationären Eingriffszahlen von 2005 bis 2017 zeigen und nach unseren Ergebnissen nur 11 % aller Eingriffe umfassen [15].

Damit handelt es sich bei den vorliegenden Daten um die erste bevölkerungsbezogene Analyse, um die möglichen Auswirkungen der veränderten Gesetzgebung zu beurteilen. Sie unterliegt jedoch einigen Limitationen. Eingriffe die außerhalb des deutschen Gesundheitssystems durchgeführt wurden, konnten in unseren Daten nicht erfasst werden. Dies ist für nicht-medizinisch indizierte Beschneidungen sowie Eingriffe außerhalb von Deutschland relevant. Außerdem verfügen wir über keine Referenzdaten aus den Jahren vor 2013, sodass es uns nur möglich ist, den unmittelbar an den Gesetzgebungszeitpunkt vom Dezember 2012 angrenzenden Zeitraum zu beschreiben. Die präsentierten Daten können wie oben beschrieben als Surrogatparameter für die Entwicklung ritueller Zirkumzisionen in Deutschland dienen. In Ermangelung belastbarer bevölkerungsbezogener Daten ermöglicht unsere Analyse erstmals eine Einschätzung der Effekte der geänderten Gesetzgebung.

## Schlussfolgerung

Mit der Verabschiedung des Beschneidungsgesetzes in Deutschland im Dezember 2012 wurde Rechtssicherheit in Bezug auf Zirkumzision bei männlichen Minderjährigen geschaffen. Zu den befürchteten deutlichen Steigerungen der Eingriffszahlen kam es jedoch nicht.

## Abkürzungen

AAP: American Academy of Pediatrics
IGeL: Individuelle Gesundheitsleistung
OPS: Operations- und Prozedurenschlüssel
